# Complement Component C4 Regulates the Development of Experimental Autoimmune Uveitis through a T Cell-Intrinsic Mechanism

**DOI:** 10.3389/fimmu.2017.01116

**Published:** 2017-09-11

**Authors:** Lingjun Zhang, Brent A. Bell, Yan Li, Rachel R. Caspi, Feng Lin

**Affiliations:** ^1^Department of Immunology, Cleveland Clinic, Cleveland, OH, United States; ^2^Cole Eye Institute, Cleveland Clinic, Cleveland, OH, United States; ^3^Laboratory of Immunology, National Eye Institute, National Institutes of Health, Bethesda, MD, United States

**Keywords:** complement, C4, autoimmune uveitis, T cells, animal models

## Abstract

In addition to its conventional roles in the innate immune system, complement has been found to directly regulate T cells in the adaptive immune system. Complement components, including C3, C5, and factor D, are important in regulating T cell responses. However, whether complement component C4 is involved in regulating T cell responses remains unclear. In this study, we used a T cell-dependent model of autoimmunity, experimental autoimmune uveitis (EAU) to address this issue. We compared disease severity in wild-type (WT) and C4 knockout (KO) mice using indirect ophthalmoscopy, scanning laser ophthalmoscopy, spectral-domain optical coherence tomography, and histopathological analysis. We also explored the underlying mechanism by examining T cell responses in *ex vivo* antigen-specific recall assays and in *in vitro* T cell priming assays using bone marrow-derived dendritic cells, splenic dendritic cells, and T cells from WT or C4 KO mice. We found that C4 KO mice develop less severe retinal inflammation than WT mice in EAU and show reduced autoreactive T cell responses and decreased retinal T cell infiltration. We also found that T cells, but not dendritic cells, from C4 KO mice have impaired function. These results demonstrate a previously unknown role of C4 in regulating T cell responses, which affects the development of T cell-mediated autoimmunity, as exemplified by EAU. Our data could shed light on the pathogenesis of autoimmune uveitis in humans.

## Introduction

Complement is an important component of the innate immune system, the primary role of which is to fight infections (1). When complement is activated through one of the three main activation pathways, i.e., the classical, alternative, and lectin pathways, membrane attack complexes are formed on target cells, resulting in direct lysis of, or damage to, the invading pathogen. At the same time, anaphylatoxins C3a and C5a are released into the fluid phase to recruit inflammatory cells to the site of complement activation and activate them to eliminate the infectious agent. In addition, the complement activation product C3b and its degradation products, iC3b and C3d, are deposited on the surface of the target cell where complement activation was initiated, facilitating phagocytosis to remove the “opsonized” target cell or pathogen. The importance of complement in the fight against invading pathogens is demonstrated by clinical findings in patients with complement deficiency or systemic complement inhibition are susceptible to opportunistic infections (2) and by experimental studies using complement gene knockout (KO) mice (3–5). The complement component C4 is essential for both the classical and lectin pathways of complement activation (6).

In addition to its critical role in the innate immune system, complement has been found to be integrally involved in regulating adaptive immunity. For example, besides opsonizing pathogens to facilitate phagocytosis, C3d also interacts with complement receptor CR2 (CD21) on B cells to significantly increase the production of antibodies against opsonized antigens (7). When hen egg lysozyme (HEL), a model antigen, was fused to two to three copies of murine C3d, a ligand of CR2, it became 1,000- to 10,000-fold more immunogenic than HEL alone in mice (8), demonstrating a direct role of complement in regulating B cells.

Complement also directly regulates T cells (9). When antigen-presenting cells (APCs) activate T cells, complement components are produced locally by both of the interacting partners and activated to generate C3a and C5a, which provide signaling to APCs and T cells to augment T cell activation and survival (9). The alternative pathway of complement activation is important in the regulation of T cells by local complement activation (10), but whether the classical and lectin activation pathways are involved in this process remains unclear. In addition, it has been shown that locally produced complement activated intracellularly is also important for T cell activation and survival (11–13).

Autoimmune uveitis, one of the most common causes of blindness, has an unknown etiology and there is no available cure (14). Polymorphisms of various complement genes have been associated with uveitis in clinical studies, but the mechanisms are not known (15–19). Experimental autoimmune uveitis (EAU) is a primarily T cell-mediated model of posterior autoimmune uveitis, in which retina-specific T cells are primed in the periphery and migrate into the eye to initiate retinal inflammation (20). In previous studies using mice deficient in C3 (21), CD55 (a cell surface complement regulator) (22), or C3aR/C5aR (receptors for C3a/C5a) (23), we and others have provided evidence suggesting that complement can directly regulate autoreactive T cell responses in EAU, but whether C4 has a similar role in regulating T cells has not been examined.

In this study, we examined a potential role of C4 in the development of EAU using wild-type (WT) and C4 KO mice. We induced EAU in sex- and age-matched WT and C4 KO mice by immunizing them with a peptide from interphotoreceptor retinoid-binding protein (IRBP) and evaluated the development and severity of EAU using various ocular imaging techniques. We also examined T cell responses using *ex vivo* T cell recall assays and carried out *in vitro* T cell activation assays using bone marrow-derived dendritic cells (BM-DCs), isolated splenic dendritic cells, and CD4+ T cells from naïve WT and C4 KO mice to further dissect the underlying mechanism involved in this process. Our results reveal a previously unknown role of C4 in regulating the autoreactive T cell responses that lead to the development of EAU.

## Reagents and Methods

### Animals

Male and female WT and C4 KO (C57BL/6J background) mice (24), aged 8–12 weeks, were obtained from Jackson Laboratory and maintained under pathogen-free conditions in the animal facilities of the Cleveland Clinic. All animal care and experimental procedures were approved by the Institutional Animal Care and Use Committee of the Cleveland Clinic and were in accordance with the U.S. Department of Health and Human Services Guide for the Care and Use of Laboratory Animals.

### Induction of EAU

Experimental autoimmune uveitis induction was performed as described previously (20). Each mouse was injected subcutaneously at multiple sites in the back and tail base with a total of 200 μl of emulsion consisting of equal volumes of *Mycobacterium tuberculosis* H37Ra-supplemented complete Freund’s adjuvant (2.5 mg/ml) (Difco Laboratories, Inc., Detroit, MI, USA) and an aqueous solution of the human IRBP_651–670_ peptide (LAQGAYRTAVDLESLASQLT) (2 mg/ml) (custom synthesized by GenScript USA Inc., Piscataway, NJ, USA). A single dose of 500 ng of pertussis toxin (List Biologic Laboratories, Inc., Campbell, CA, USA) was injected intraperitoneally on the same day.

### Clinical and Histopathological Scoring

After immunization, clinical manifestations were examined daily using a binocular indirect ophthalmoscope (Keeler Instruments, Inc., Broomall, PA, USA). The pupils were dilated with a mixed ophthalmic solution of 0.5% tropicamide and 1.25% phenylephrine hydrochloride and disease severity was scored on a scale of 0–4, according to published criteria (20).

On day 21 after immunization, the mice were euthanized. For histopathological evaluation, whole eyes were collected and fixed in 10% formaldehyde/PBS buffer for 24 h and the fixed tissues embedded in paraffin. Sections (5 µm) were cut through the pupil and optic nerve axis and processed for H&E staining, then retinal histopathological changes were graded on a scale of 0–4 according to previously published scoring criteria (20).

### Retinal Imaging Analyses

Retinal imaging was performed as described previously (23) using cSLO, SD-OCT, and TEFI under general anesthesia. A cSLO (Heidelberg Retina Angiograph II; Heidelberg Engineering, Carlsbad, CA, USA) was used to collect both reflectance and fluorescence information from the posterior segment. The SD-OCT system used was a 840 HR SDOIS (Bioptigen, Inc., Morrisville, NC, USA) with a central operating wavelength of ~840 nm and an in-depth, axial resolution of ~6 μm. Conventional visible light fundus images were collected using a custom-fabricated TEFI apparatus (25). Number of hyper-reflective foci in vitreous chamber of OCT images areas were quantitated using ImageJ software (http://imagej.nih.gov/ij/, National Institutes of Health, Bethesda, MD, USA).

### *Ex Vivo* T Cell Recall Assays

T cell recall assays were performed on day 21. For each of the immunized WT and C4 KO mice, 4 × 10^5^ splenocytes were cultured in 96-well round-bottomed microtiter plates in 100 µl of complete RPMI 1640 medium in the presence or absence of 20 µg/ml of peptide IRBP_651–670_ or a non-relevant peptide (ovalbumin OVA_323–339_, Genscript, NJ, USA) for 72 h, then the supernatants were collected and interferon (IFN)γ and interleukin (IL)-17 levels measured using ELISA kits (BioLegend, San Diego, CA, USA).

### BM-DC Differentiation Assay

Following a previously established protocol (26), BM cells were harvested from the femur of WT and C4 KO mice and cultured for 48 h in complete medium containing 10 ng/ml of granulocyte-macrophage colony-stimulating factor (GM-CSF) and 100 U/ml of IL-4 (Peprotech, Rocky Hill, NJ, USA), then, non-adherent cells were gently removed and the medium replaced with fresh medium containing the same concentrations of GM-CSF and IL-4. Double-positive cells (DCs) were harvested on day 5 and DC differentiation was assessed by counting MHC II and CD11c DCs using a flow cytometer.

### Differentiated DC Function Assay

The function of the differentiated DCs was compared by measuring their ability to activate antigen-specific T cells. In brief, T cells were enriched from splenocytes of OTII mice (Jackson Laboratory) using nylon wool and labeled with carboxyfluorescein succinimidyl ester (CFSE) according to the manufacturer’s instruction (Thermo Fisher Scientific, Waltham, MA, USA), then were cocultured with the same numbers of differentiated WT or C4 KO DCs (10:1 ratio) in the presence or absence of 2 μg/ml of peptide OVA_323–339_ for 72 h. The proliferation of the activated T cells was then measured by CFSE dilution (27) using flow cytometry, while IFNγ production by the activated T cells were measured by analyzing the culture supernatants using ELISA (Biolegend) following the manufacturer’s recommended protocol.

### Splenic DCs Isolation and Function Assay

Splenic DCs from WT and C4 KO mice were isolated using a mouse pan-DC enrichment kit (Stemcell Technologies Inc., Cambridge, MA, USA) following the manufacturer’s instructions. The isolated splenic DCs were matured with 2 µg/ml LPS 4 h before being cocultured with CFSE-labeled CD4+ T cells isolated from OTII mouse splenocytes using a mouse CD4+ T cell enrichment kits (Stemcell Technologies Inc.) at a ratio of 10:1 (T cells: DCs) in the absence or presence of 2 µg/ml of OVA_323–339_ peptide for 72 h. CD4+ T cells isolated by this protocol usually reaches >97% purity based on flow cytometric analysis. The proliferation of the DC-activated T cells was analyzed by flow cytometry, and levels of IFNγ produced by the activated T cells in the culture supernatants were measured using ELISA.

### *In Vitro* T Cell Activation, Proliferation, and Apoptosis Assays

CD4+ T cells were isolated as described above according to the manufacturer’s instructions, then, 4 × 10^5^ of the purified T cells were seeded in 96-well round-bottomed microtiter plates precoated overnight with 5 µg/ml of anti-mouse CD3 mAb (Biolegend, CA, USA) in the presence of 1 µg/ml of anti-mouse CD28 mAb and 1,000 U/ml of mouse IL-2 (Peprotech, Rocky Hill, NJ, USA).

For the activation assay, T cells were collected and stained with a PE-labeled anti-CD69 mAb for 5 h. For the T cell proliferation assay, 10 nM BrdU was added to the plates for 48 h, then incorporated BrdU was detected using a BrdU ELISA kit (BD Biosciences, San Jose, CA, USA) after DNase treatment according to the manufacturer’s protocol. For the apoptosis assay, cells were collected and stained with Annexin V and PI as recommended by the manufacturer (BD Biosciences, San Jose, CA, USA).

### Statistical Analysis

GraphPad Prism 6 (GraphPad Software, Inc., La Jolla, CA, USA) was used for data analysis. Data were expressed as means ± SEM unless labeled otherwise. The EAU clinical scores were assessed by two-way ANOVA. Two sets of data were compared by Student’s *t*-test and three sets of data were analyzed by one-way ANOVA. *p*-Values less than 0.05 were considered significant.

## Results

### C4 KO Mice Exhibit Lower EAU Clinical Scores and Retinal Histopathological Scores than WT Mice after Immunization

**Figure 1:**
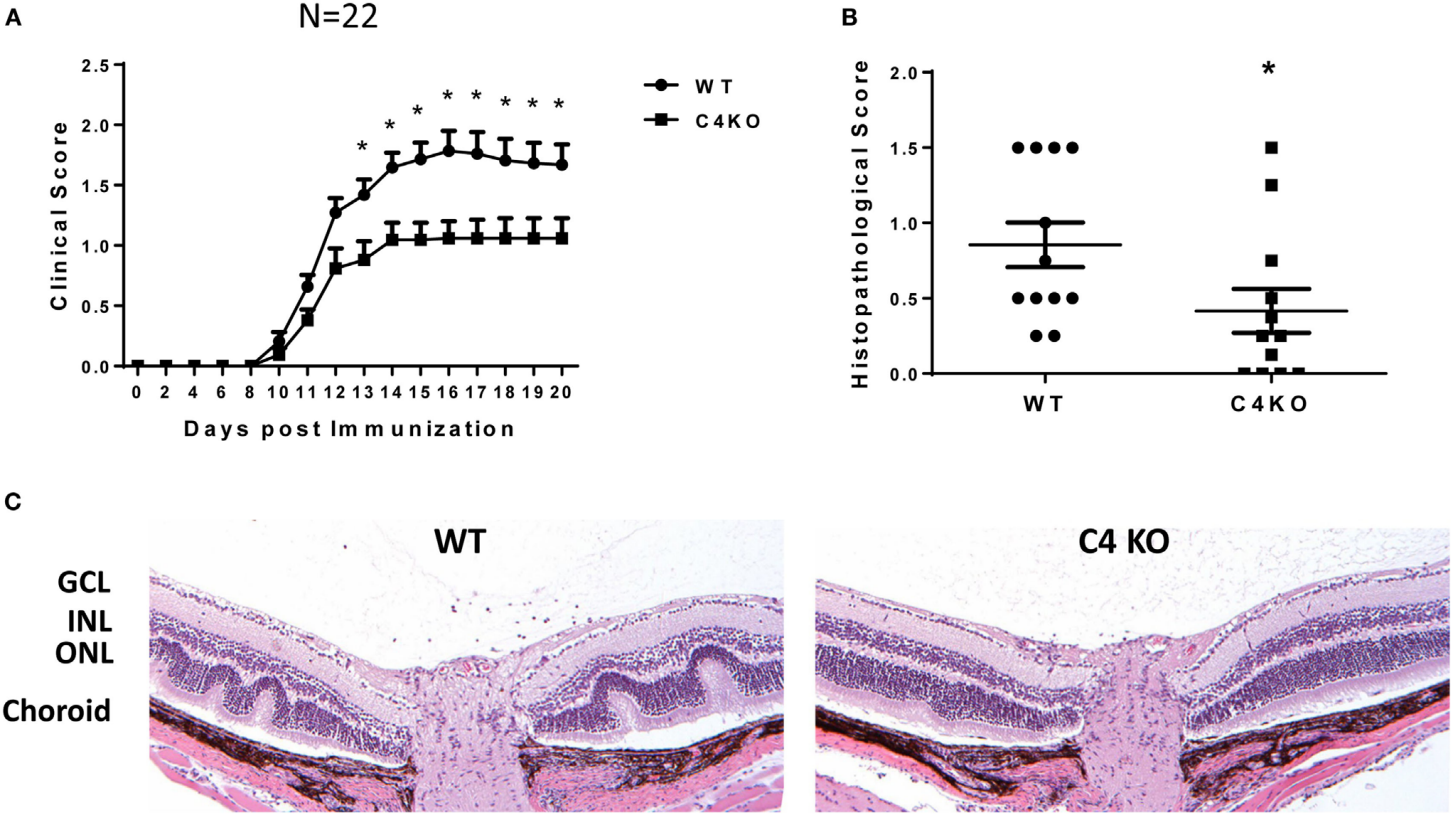
C4 KO mice show reduced disease severity in experimental autoimmune uveitis (EAU). **(A)** EAU clinical scores of wild-type (WT) and C4 KO mice after immunization to induce EAU (*n* = 22 in each group, mean ± SEM). Age- and sex-matched WT and C4 KO mice were immunized with interphotoreceptor retinoid binding protein peptide to induce EAU, then their eyes were examined by indirect ophthalmoscopy daily from day 7 to day 21 and the clinical score recorded. **(B)** EAU histopathological scores for the WT and C4 KO mice at day 21 after immunization to induce EAU (*n* = 12) mean ± SEM. **(C)** Representative histological analysis of retina sections from WT and C4 KO mice at day 21 after immunization. GCL, the ganglion cell layer; INL, the inner nuclear layer; ONL, the outer nuclear layer. *p < 0.05 by two-way ANOVA or unpaired *t*-test.

To explore the potential role of C4 in the pathogenesis of EAU, we first immunized age- and sex-matched WT and C4 KO mice with IRBP peptide in CFA to induce EAU following a previously published protocol. We followed the development of EAU daily by indirect ophthalmoscopy from day 7 to day 20 after immunization, then euthanized the mice to prepare ocular sections for histopathological scoring and other assays. We found that, compared to clinical scores of ~1.5–1.8 in WT mice at day 14 after EAU induction, those in the C4 KO mice were significantly lower, with an average score of ~1.0 (Figure 1A) and that histopathological scores for the ocular sections on day 21 were also significantly lower in the C4 KO mice (0.41 ± 0.15 vs 0.85 ± 0.15) (Figures 1B,C).

### C4 KO Mice Develop Milder Retinal Pathological Features after EAU Induction

**Figure 2:**
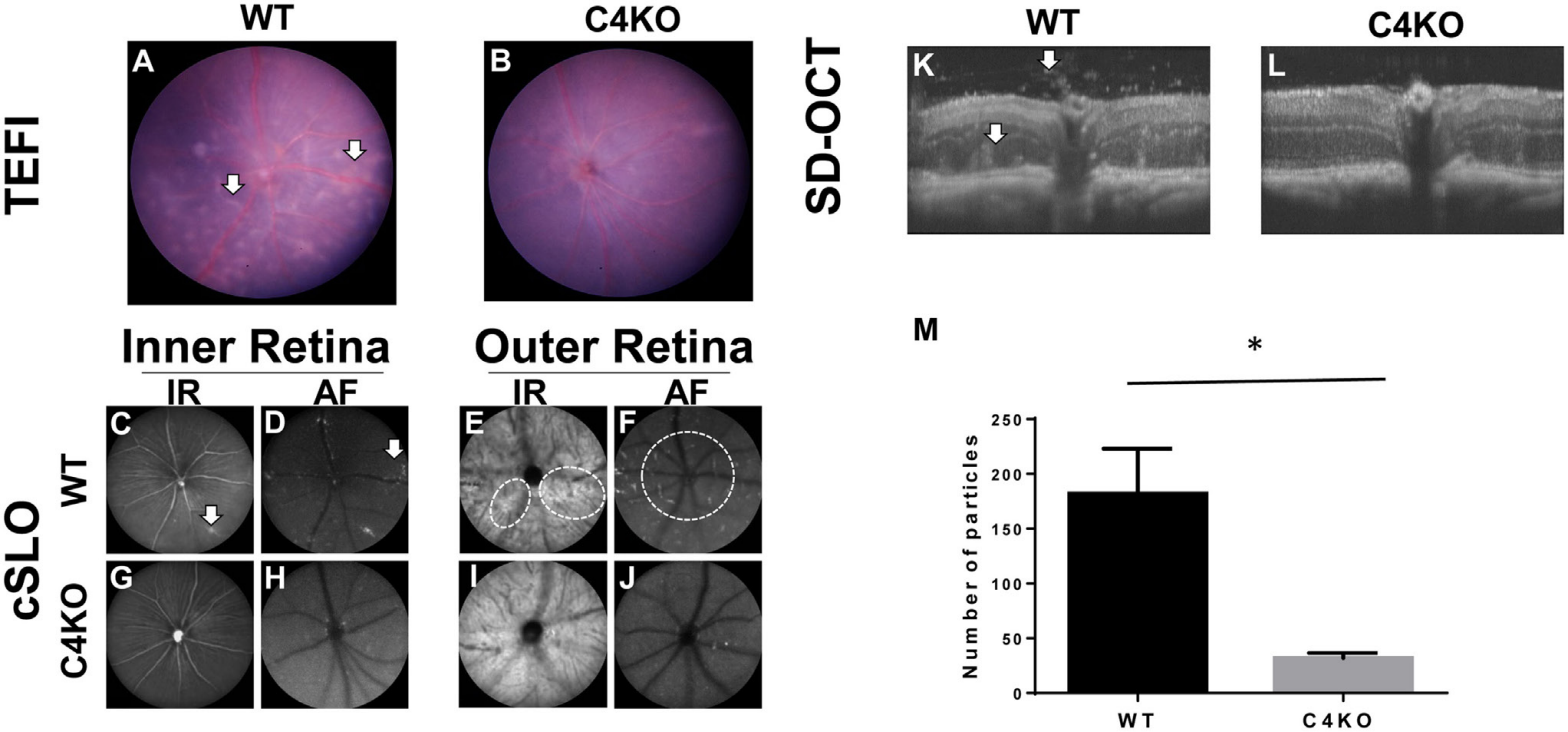
Representative pictures in wild-type (WT) and C4 KO mice with experimental autoimmune uveitis at day 15 after immunization using different ocular imaging techniques. **(A,B)** TEFI imaging showed linear and confluent chorioretinal lesions were common in WTs [**(A)**, arrows] but significantly less in C4 KOs **(B)**; **(C–J)** cSLO images showed hyper-reflective features to both inter **(C,D,G,H)** and outer layers **(E,F,I,J)** of the retina. Numerous foci in the inter retina can be found adjacent to retinal vessels in the WTs [**(C,D)**, arrows]. These foci were significantly fewer in the KOs **(G,H)**. In the outer retina, other hyper-reflective features emanating from or around the optical nerve can be seen in the WTs [**(E,F)**, circles] but rarely found in the KOs **(I,J)**; **(K,L)** SD-OCT images revealed more reflective foci and retinal infoldings in the WTs [**(K)**, arrows] than with those in the KOs **(L)**. A summary of the numbers of hyper-reflective foci in vitreous cavities was presented in panel **(M)**.

In addition to the above tests, we used three ocular imaging techniques, TEFI, cSLO, and SD-OCT, to evaluate the severity of EAU in the WT and C4 KO mice on day 15 after disease induction. These studies showed multiple retinal pathologic changes in the WT mice, including focal and linear chorioretinal lesions observed by TEFI (Figure 2A) and increased hyper-reflective features adjacent to the retinal vessels in the inner retina and around the optic nerve in the outer retina observed by cSLO (Figures 2C–F). SD-OCT also revealed inflammatory cell infiltration in the vitreous chamber, retinal infoldings, and a disrupted inner segment/outer segment band in the WT mice (Figure 2K). All of these features were much less obvious in the C4 KO mice (Figures 2B,G–J,L). Hyper-reflective foci in vitreous cavity are likely cells and/or cellular aggregates that have infiltrated into the vitreous. We quantitated these foci and found that C4 KO mice showed signficantly lower numbers of these foci in the vitreous cavity than WT mice in EAU (*p* = 0.003, Figure 2M).

### C4 KO Mice Develop Lower IBRP-Specific Th1 and Th17 Responses

**Figure 3:**
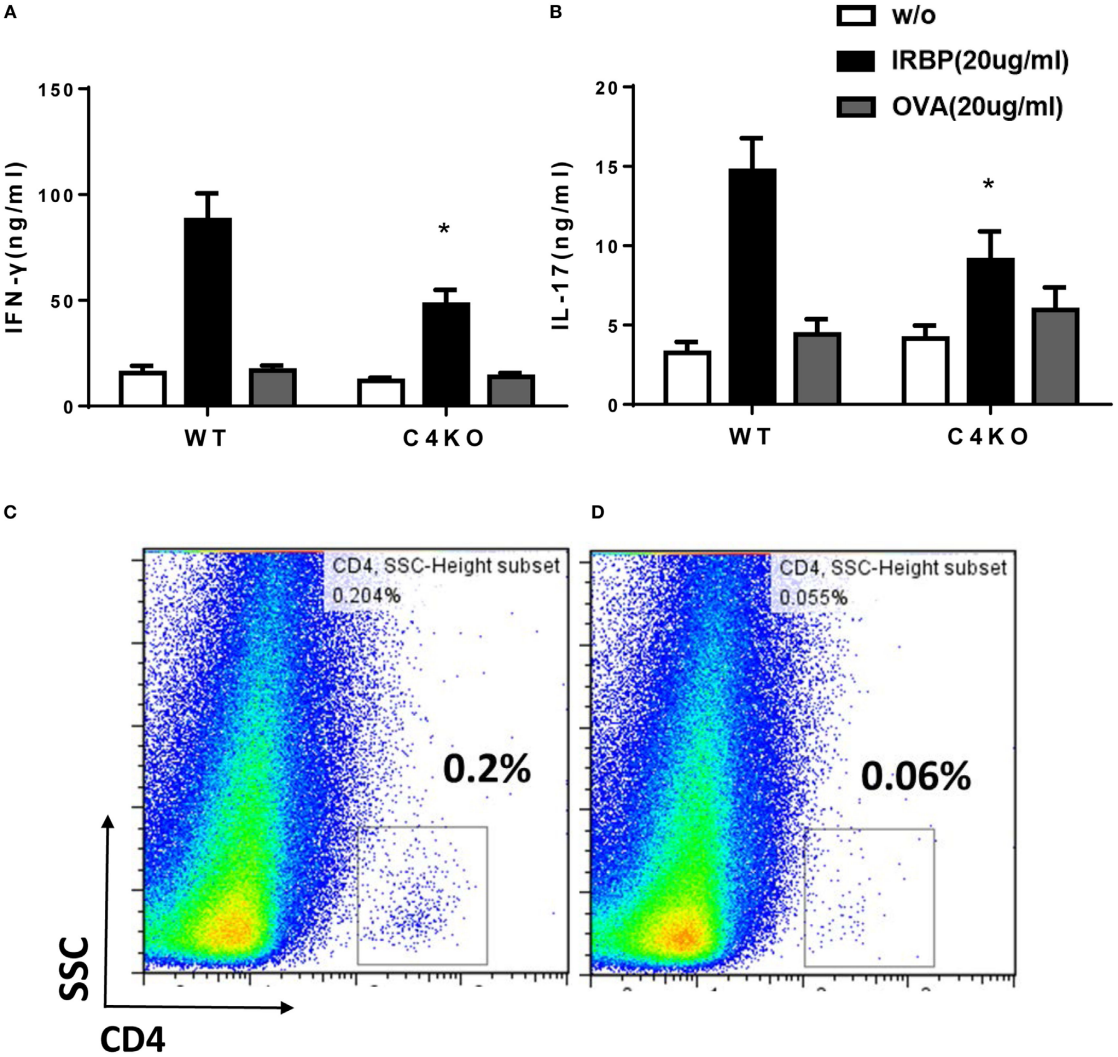
*Ex vivo* immunological studies of wild-type (WT) and C4 KO mice in experimental autoimmune uveitis (EAU). At day 21 after EAU induction, splenocytes were collected from WT and C4 KO mice and cultured for 72 h without antigen (w/o) or with 20 µg/ml of the immunizing interphotoreceptor retinoid binding protein (IRBP) peptide or a non-relevant peptide (OVA), then, levels of interferon (IFN)γ **(A)** and IL-17 **(B)** were measured by ELISA (*n* = 22 in each group) mean ± SEM; *p < 0.05 by one-way ANOVA. On the same day, eyes were collected from five mice in each group and single cell suspensions prepared and pooled, then the percentage of CD4+ T cells in the infiltrating cells in the eye of WT **(C)** and C4 KO **(D)** mice was analyzed by flow cytometry.

We used *ex vivo* antigen-specific recall assays to determine pathogenic Th1 and Th17 responses after EAU induction. We collected splenocytes from WT and C4 KO mice on day 21, then incubated the cells with or without 20 µg/ml of IRBP peptide or a non-relevant peptide, OVA_323–339_, for 72 h, then measured levels of IFNγ and IL-17 in the culture supernatants by ELISA. These *ex vivo* antigen-specific T cell recall assays showed that IRBP-specific T cells from immunized C4 KO mice produced only 54.21 ± 8.58 ng/ml of IFNγ and 9.10 ± 1.81 ng/ml of IL-17 compared to 94.63 ± 15.96 ng/ml of IFNγ and 14.72 ± 2.05 ng/ml of IL-17 produced by cells from WT mice (Figures 3A,B). These data suggest that pathogenic Th1 and Th17 responses were significantly reduced in C4 KO mice compared to WT mice in EAU.

### C4 KO Mice with EAU Have Reduced Numbers of Ocular-Infiltrating CD4+ T Cells than WT Mice with EAU

Since ocular infiltration of lymphocytes and other inflammatory cells is a hallmark of EAU, we compared numbers of ocular-infiltrating CD4+ cells in WT and CD4 KO mice with EAU. In brief, at day 21 after immunization, we treated enucleated eyes from five mice from each group with 1 mg/ml collagenase, then pooled the resultant single cells together and incubated them with an anti-CD4 mAb and analyzed the stained cells by flow cytometry. As shown in Figures 3C,D, 0.2% of the cells from the eyes of WT mice with EAU were CD4+, but this value was only 0.06% in the eyes of CD4 KO mice with EAU, showing that C4 KO mice have reduced ocular T cell infiltration in EAU.

### DCs from Naïve C4 KO Mice and WT Mice Stimulate Similar Antigen-Specific T Cell Responses

**Figure 4:**
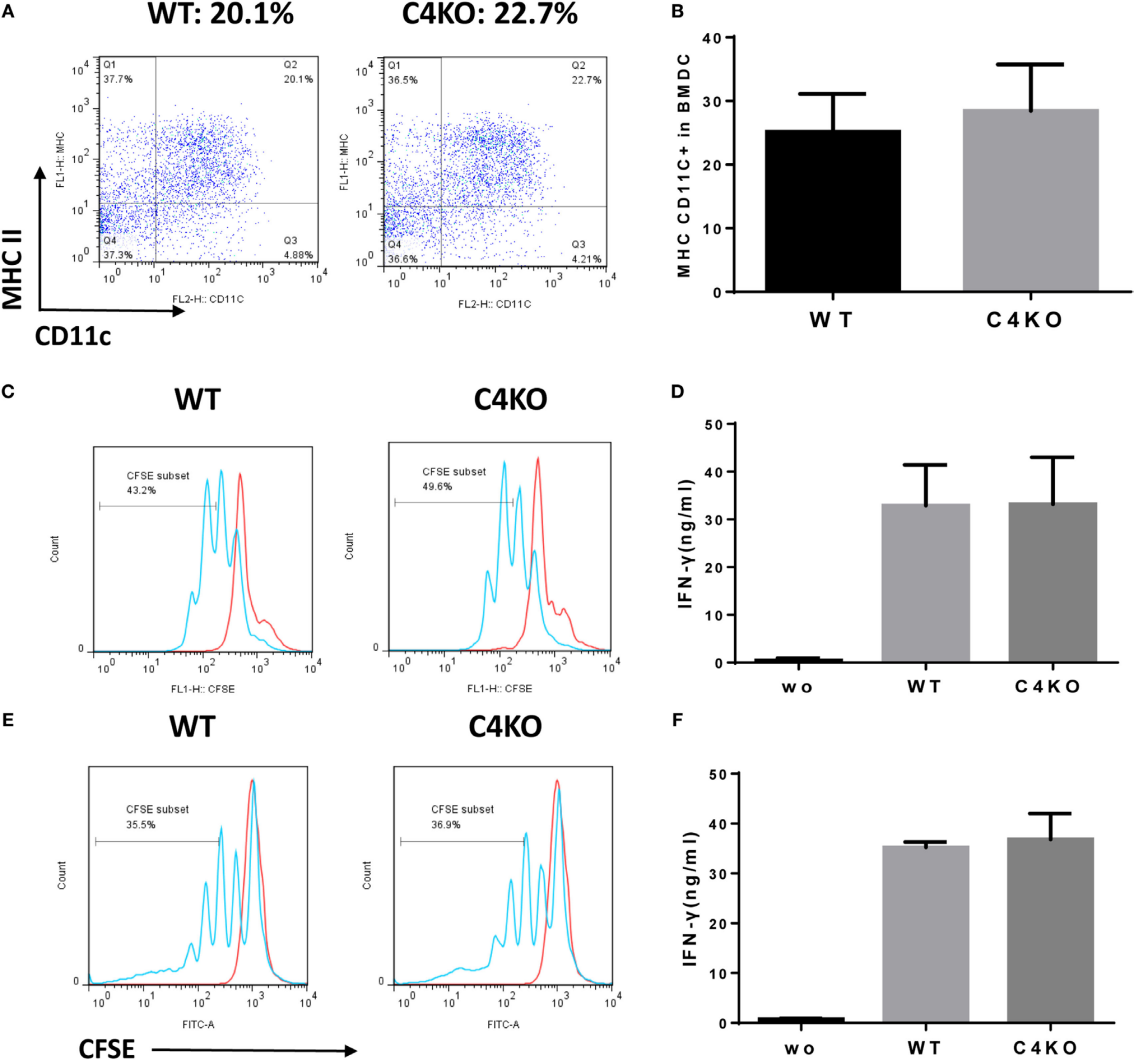
Effects of C4 deficiency on DC differentiation and function. BM cells from naïve wild-type (WT) and C4 KO mice were cultured under DC differentiation conditions and differentiated double-positive cells (DCs) (MHCII+ CD11C+) analyzed by flow cytometry. **(A,B)** Representative flow cytometric analysis results for WT and C4 KO DC differentiation **(A)** and the summarized results **(B)** [*n* = 5 in each group, mean ± SEM]. **(C,D)** The same numbers of BM-derived WT or C4 KO DCs was then cultured with carboxyfluorescein succinimidyl ester (CFSE)-labeled T cells from OTII mice in the presence or absence (wo) of peptide OVA_323–339_ for 72 h, then the proliferation of the activated T cells was assessed by flow cytometry **(C)** and levels of interferon (IFN)γ produced by the activated T cells measured by ELISA **(D)** (*n* = 5 in each group). The same T cell proliferation **(E)** and IFNγ **(F)** assays were done using splenic DCs isolated from WT and C4 KO mice (*n* = 3 in each group), no statistically significant difference was observed either (mean ± SEM).

Our *in vivo* and *ex vivo* studies above show that pathogenic T cell responses were reduced in EAU C4 KO vs EAU WT mice. Since DCs are known to locally produce C4 (28), it is possible that absence of C4 in DCs could impair DC differentiation and/or the function of the differentiated DCs. To explore the mechanism underlying attenuated pathogenic T cell responses in C4 KO mice with EAU, we generated DCs from the BM of naïve WT or C4 KO mice and assessed the differentiation of DCs by quantifying the resultant MHC II/CD11c DCs using flow cytometry and found no significant difference (Figures 4A,B). We then incubated the same numbers of the differentiated WT or C4 KO DCs with the CFSE-labeled T cells from naïve OTII mice in the presence or absence of peptide OVA_323–339_ for 72 h, then measured T cell proliferation by flow cytometry and IFNγ levels in the culture supernatants by ELISA. As shown in Figure 4C, OTII T cells cocultured with WT or C4 KO DCs showed similar proliferation after OVA stimulation. In addition, Figure 4D shows that, while IFNγ was barely detectable in DC/OTII T cell cocultures in the absence of OVA peptide, comparable IFNγ levels was produced by T cells stimulated with either WT or C4 KO DCs. In addition to the BM-derived DCs, we also studied the splenic DCs from WT and C4 KO mice and got similar results (Figures 4E,F). These data suggest that C4 deficiency in DCs is not causally involved in the reduced antigen-specific T cell response seen in EAU C4 KO mice.

### CD4+ T Cells from Naïve C4 KO Mice Have Impaired Function

**Figure 5:**
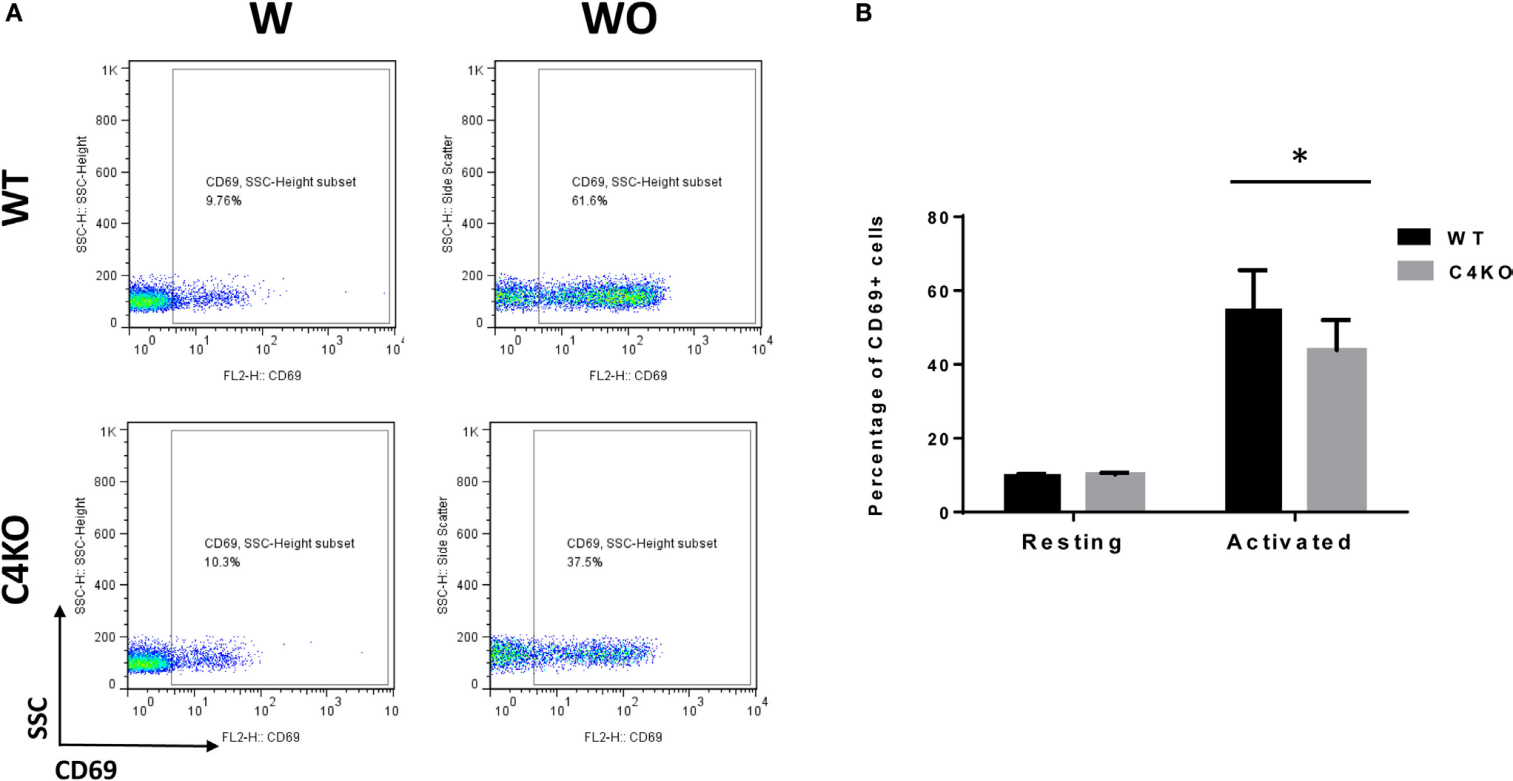
Effect of C4 deficiency on T cell activation. CD4+ T cells purified from naïve wild-type (WT) and C4 KO mice were incubated with (w) or without (w/o) anti-CD3 and anti-CD28 mAbs for activation for 5 h, then the percentage of activated T cells (CD69+) in the total cells was quantitated by flow cytometry. **(A)** Representative flow cytometric analysis results. **(B)** Summarized results from three independent experiments. Mean ± SEM. *p < 0.05 by paired *t*-test.

**Figure 6:**
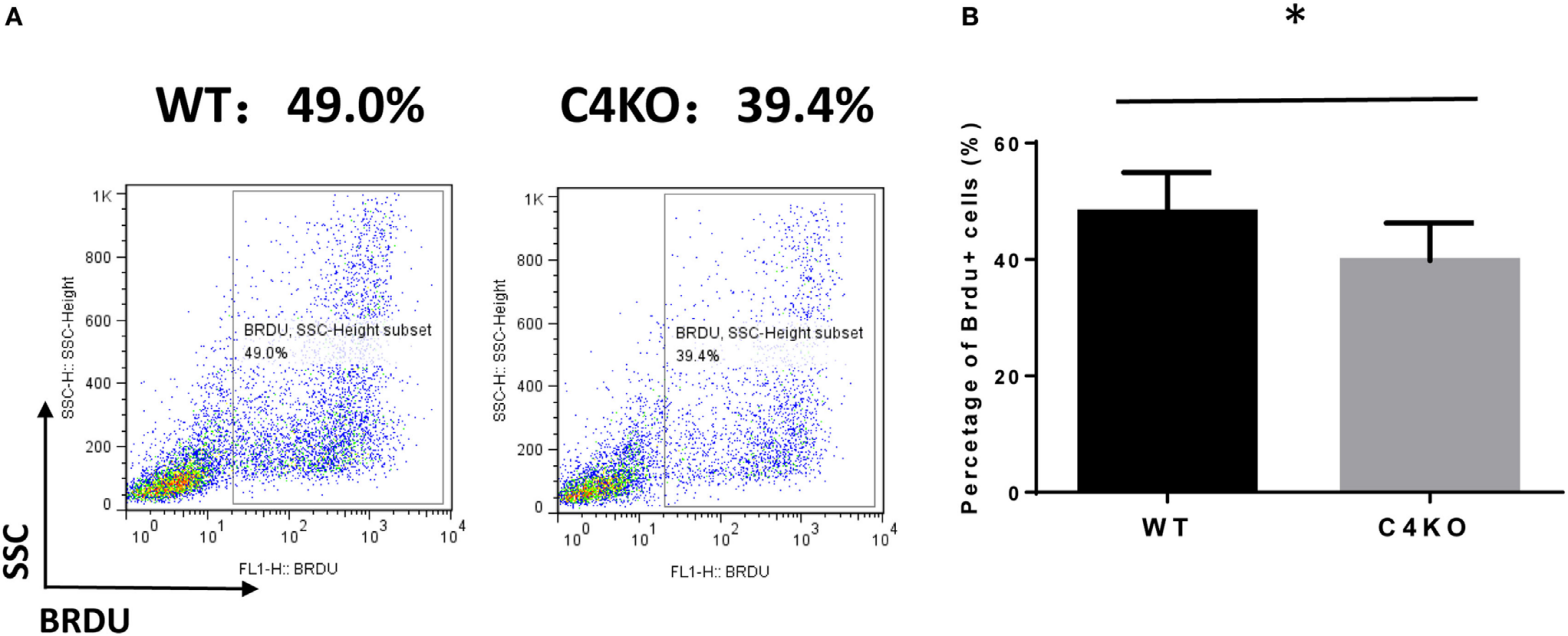
Effect of C4 deficiency on the proliferation of activated T cells. CD4+ T cells purified from naïve wild-type (WT) and C4 KO mice were incubated for 24 h with anti-CD3 and anti-CD28 mAbs and BrdU, then the proliferation of the activated T cells was assessed by estimating the percentage of BrdU+ T cells in the total cells using flow cytometric analysis. **(A)** Representative results. **(B)** Summarized results from three independent experiments. Mean ± SEM. *p < 0.05 by paired *t*-test.

**Figure 7:**
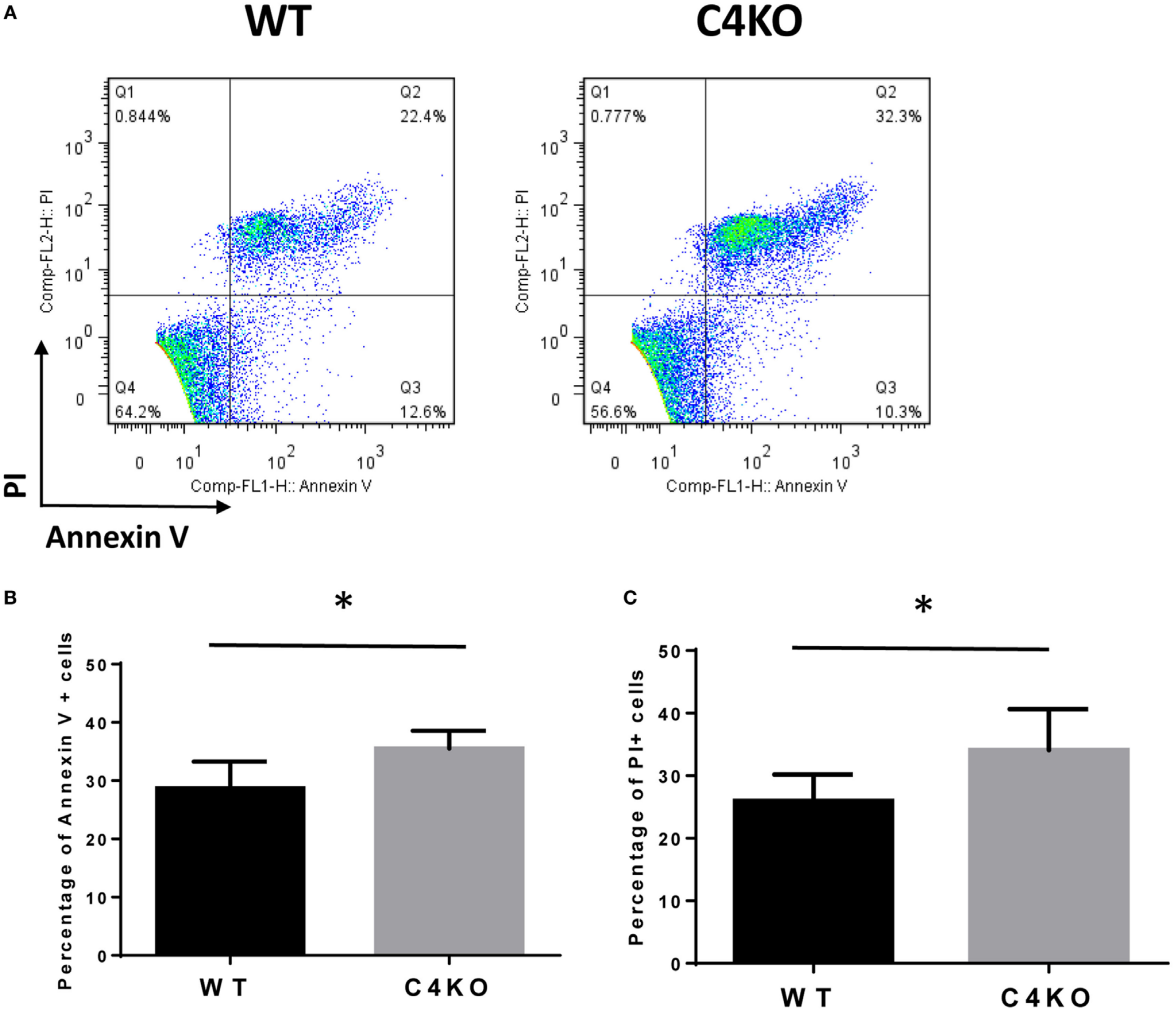
Effect of C4 deficiency on the survival of activated T cells. CD4+ T cells purified from naïve wild-type (WT) and C4 KO mice were incubated with anti-CD3 and anti-CD28 mAbs for 48 h, then the percentage of apoptotic or dead T cells was assessed, respectively, by Annexin V or PI staining, followed by flow cytometric analysis. **(A)** Representative results; **(B,C)** summarized results from three independent experiments. Mean ± SEM. *p < 0.05 by paired *t*-test.

**Figure 8:**
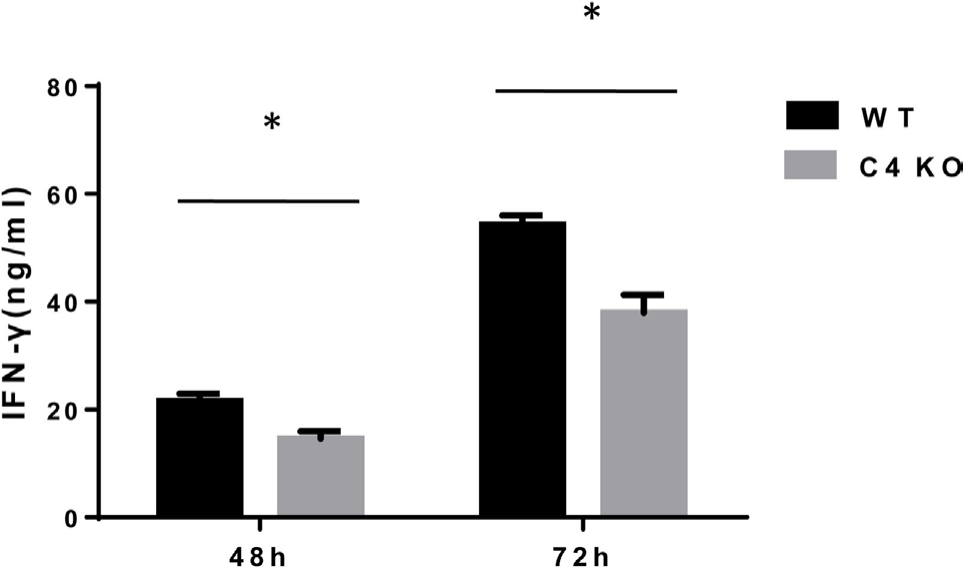
Effect of C4 deficiency on cytokine production by activated T cells. CD4+ T cells purified from naïve wild-type (WT) and C4 KO mice were incubated for 48 or 72 h with anti-CD3 and anti-CD28 mAbs, then interferon (IFN)γ levels in the culture supernatants were measured by ELISA after 48 or 72 h. *n* = 3 in each group, mean ± SEM. *p < 0.05 by *t*-test.

To assess the impact of C4 deficiency from the T cell side, we purified CD4+ T cells from naïve WT or C4 KO mice, then activated them with anti-CD3 and anti-CD28 mAbs, together with IL-2, then evaluated T cell activation after 5 h by comparing levels of the T cell activation marker, CD69, and investigated the proliferation of the activated T cells after 24 h (BrdU incorporation) and their survival after 48 h (Annexin V and PI staining). Finally, we measured IFNγ levels in the culture supernatants after 48 and 72 h by ELISA. Figures 5A,B show that, after 5 h of activation, CD69 levels were significantly lower on C4 KO T cells than on WT T cells. In addition, the proliferation of activated C4 KO T cells was reduced, as indicated by a significantly lower percentage of BrdU+ cells in the C4 KO T cells than in the activated WT T cells at 24 h (Figures 6A,B), and there were significantly more apoptotic (Annexin V+) and dead (PI+) C4 KO T cells than WT T cells at 48 h after activation (Figure 7). Finally, activated WT T cells produced higher levels of IFNγ than activated C4 KO T cells at both 48 and 72 h (48 h: 21.56 ± 1.39 vs 14.63 ± 1.36, 72 h: 54.33 ± 1.73 vs 37.94 ± 3.34 ng/ml) (Figure 8). These results demonstrate that T cells from naïve C4 KO mice show reduced activation, proliferation, survival, and inflammatory cytokine production, which could explain the decreased IRBP-specific T cell responses in C4 KO mice in EAU.

## Discussion

In this study, we examined the potential role of C4 in EAU by comparing disease severity in age- and sex-matched WT and C4 KO mice after EAU induction using various ocular imaging, histopathological, and immunological analyses. Overall, we found that mice lacking C4 exhibited a milder EAU response than the WTs. In accordance with the attenuated ocular inflammation seen in the C4 KO mice after immunization, our *ex vivo* IRBP-specific recall experiments and ocular CD4+ T cell infiltration analysis showed reduced development of IRBP-specific IFNγ- and IL-17-producing T cells and decreased numbers of infiltrating CD4+ cells in the eyes of C4 KO mice in EAU. The *in vitro* mechanistic studies showed that DC differentiation in naïve WT and C4 KO mice was similar and that the differentiated DCs from naïve WT and C4 KO mice had a comparable ability to activate antigen-specific T cells. Interestingly, CD4+ T cells from naïve C4 KO mice showed attenuated activation and reduced proliferation, survival, and inflammatory cytokine production after activation.

The role of C4 in autoimmune diseases is paradoxical. While the majority (>75%) of patients deficient in C4 develop lupus-like autoimmune diseases, some of which are associated with autoimmune uveitis (29, 30), suggesting that C4 is protective, it has also been reported that C4 levels are significantly elevated in patients with active Behçet’s disease (BD) (31), another autoimmune disease that frequently involves uveitis, compared to BD patients in remission or non-BD controls, suggesting that C4 is pathogenic. It has also been found that, although C4 deficiency in mice leads to the development of autoantibodies (32), paradoxically, these C4 KO mice also show lower B cell responses after virus infection or immunization (33, 34), potentially due to impaired stimulation of memory B cell responses. Interestingly, although a role for C4 in the humoral response in mice seems to have been established, whether C4 directly regulates T cells remains unclear. Our *ex vivo* antigen-recall assay and T cell activation assay results show, for the first time, that C4 is required for normal T cell activation, proliferation, and survival.

Our results showing that C4 KO mice develop milder EAU, a primarily T cell-dependent autoimmune disease model, than WT mice based on various ocular imaging examinations and retinal histopathological analyses demonstrate a previously unappreciated role of C4 in the development of EAU and, potentially, autoimmune uveitis. These results, together with previous reports by ourselves and others showing that complement components accumulated adjacent to sites of inflammation in EAU (35), that C3 KO mice (21) and C3aR/C5aR KO mice (23) develop less severe EAU than WT mice, and that treating WT mice with complement inhibitors, such as recombinant DAF (22), soluble Crry (21), or an anti-C5 mAb (36), reduces the severity of EAU, confirm a critical role of the complement system in the pathogenesis of EAU and raise the possibility that it may influence its human counterpart, autoimmune uveitis.

Complement activation by the alternative pathway has been shown to directly regulate T cell responses (10). Factor B KO mice are resistant to induction of experimental autoimmune encephalomyelitis (EAE), a primarily T cell-mediated model of multiple sclerosis, and show reduced T cell responses (37), inhibition of the alternative pathway using an anti-factor B mAb suppresses T cell responses in experimental autoimmune anterior uveitis in rats (38), and, more directly, splenocytes from factor D KO mice stimulate significantly reduced T cell responses in a mixed lymphocyte reaction assay (10). Even though the role of the alternative pathway of complement activation in regulating T cell responses has been well established, whether the other two pathways, the classical and the lectin pathways, also play a role in regulating T cells was not known.

C4 is critical for both the classical and lectin pathways of complement activation. In humans, C4 gene copy number variance, polymorphisms, and blood concentrations of C4 protein have been associated with autoimmune diseases (39), including systemic lupus erythematosus, a disease in which both autoreactive T and B cells are integrally involved in the pathogenesis (40). In agreement with clinical study results, C4 KO mice have been found to have a profound deficit in antibody responses against a T cell-dependent antigen (41) and are also resistant to induction of experimental autoimmune myasthenia gravis (42), a T cell-dependent and B cell-mediated disease. However, similar disease severity was reported between C4 KO mice and WT mice after immunization with peptide MOG_35–55_ to induce EAE (43), suggesting that C4 is not involved in regulating autoreactive T cells in this model; unfortunately, no direct T or B cell assays were described in this report.

C4 can be locally produced by different kinds of APCs, including DCs (28), monocytes (44), and macrophages (45), suggesting that locally produced C4, like other complement components locally produced by APCs, e.g., factor D, C3, and C5, could be integrally involved in regulating T cells. However, our results showing that C4 KO and WT DCs stimulated similar levels of inflammatory cytokine production by antigen-specific T cells do not support this hypothesis. In fact, our results using WT and C4 KO DCs are consistent with those in previous studies showing that WT and C4 KO macrophages stimulate comparable levels of proliferation of, and IFNγ production by, WT T cells in a mixed lymphocyte reaction assay (46), suggesting that C4 locally produced by APCs is not essential for efficient T cell activation.

In addition to APCs, T cells also locally produce some complement components, such as C3, to directly regulate T cell responses (47). However, we failed to detect any C4 transcript in purified mouse T cells by RT-PCR (data not shown) and there is no evidence in the literature that T cells locally produce C4. Nevertheless, we found that T cells from naïve C4 KO mice showed reduced activation and decreased proliferation, survival, and IFNγ production after activation compared to T cells from naïve WT mice. These results suggest that T cells from C4 KO mice might be “educated” indirectly by the absence of C4 *in vivo*. How T cells are “educated” differently *in vivo* in the absence of C4 warrants further study.

In summary, by studying EAU development in WT and C4 KO mice, we found that retinal inflammation was attenuated and autoreactive T cell responses reduced in the absence of C4 using various ocular imaging, histopathological and immunological assays. Our mechanistic studies suggest that lack of C4 on DCs has no direct effect on the regulation of autoreactive T cell responses, but its absence on other cells might do so indirectly by an effect on the “education” of T cells.

## Author Contributions

LZ, BB, and YL performed the experiments, analyzed data, and edited the manuscript. RC analyzed data and reviewed/edited the manuscript. FL designed the experiments, analyzed the data, and prepared the manuscript.

## Conflict of Interest Statement

The authors declare that the research was conducted in the absence of any commercial or financial relationships that could be construed as a potential conflict of interest.
