# Supplementary figures and images for: Complement Component C4 Regulates the Development of Experimental Autoimmune Uveitis through a T Cell-Intrinsic Mechanism
Source: Front Immunol. 2017 Sep 11;8:1116. doi: 10.3389/fimmu.2017.01116 (PMC5601957; doi:10.3389/fimmu.2017.01116)

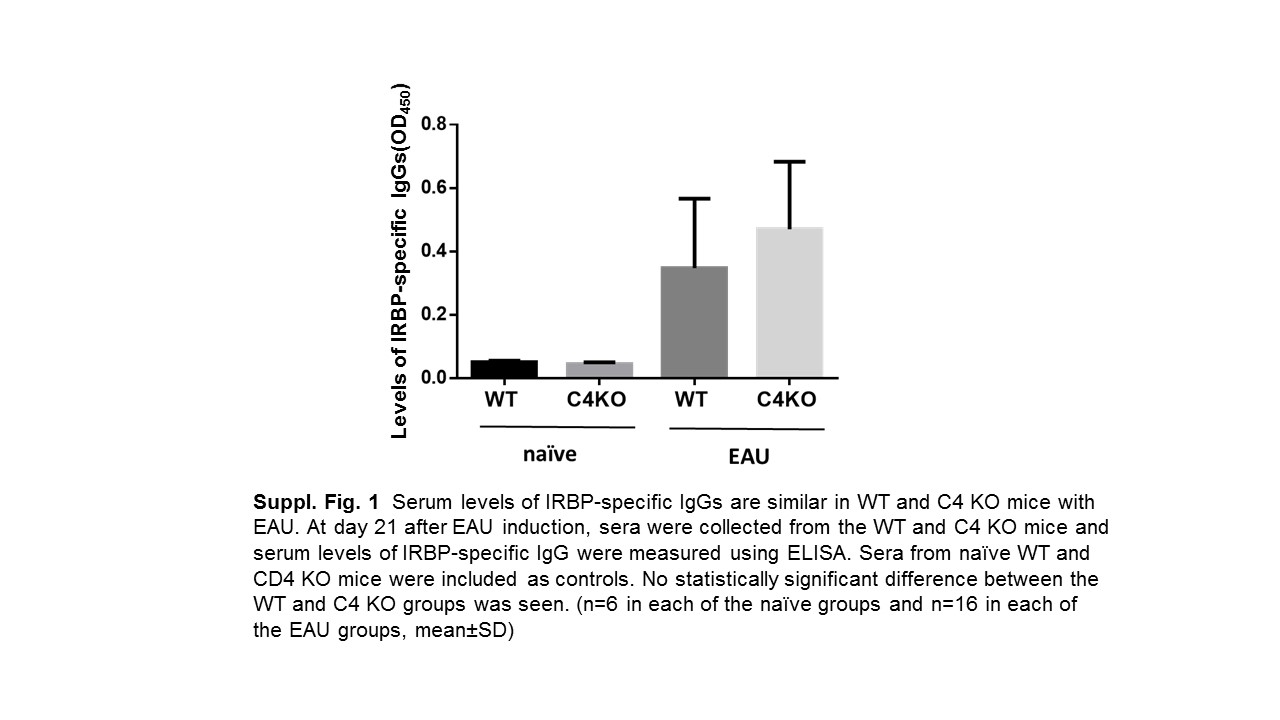

Supplement: Supplementary file 1 [file image_1.jpg]
